# The *Fusarium* metabolite culmorin suppresses the in vitro glucuronidation of deoxynivalenol

**DOI:** 10.1007/s00204-019-02459-w

**Published:** 2019-05-02

**Authors:** Lydia Woelflingseder, Benedikt Warth, Immina Vierheilig, Heidi Schwartz-Zimmermann, Christian Hametner, Veronika Nagl, Barbara Novak, Bojan Šarkanj, Franz Berthiller, Gerhard Adam, Doris Marko

**Affiliations:** 1 Department of Food Chemistry and Toxicology, Faculty of Chemistry, University of Vienna, Währingerstrasse 38, 1090 Vienna, Austria; 2 Christian Doppler Laboratory for Mycotoxin Metabolism and Center for Analytical Chemistry, Department of Agrobiotechnology (IFA-Tulln), University of Natural Resources and Life Sciences, Vienna (BOKU), Konrad-Lorenz-Strasse 20, 3430 Tulln, Austria; 3 Institute of Applied Synthetic Chemistry, Vienna University of Technology, Getreidemarkt 9/163, 1060 Vienna, Austria; 4 BIOMIN Research Center, Technopark 1, 3430 Tulln, Austria; 5 Department of Applied Chemistry and Ecology, Faculty of Food Technology, Josip Juraj Strossmayer University of Osijek, Franje Kuhača 20, 31000 Osijek, Croatia; 6 Department of Applied Genetics and Cell Biology, University of Natural Resources and Life Sciences, Vienna (BOKU), Konrad-Lorenz-Strasse 24, 3430 Tulln, Austria

**Keywords:** Natural contaminants, Drug metabolism, Fungal metabolites, Chemical mixtures, Drug–exposome interactions

## Abstract

Glucuronidation is a major phase II conjugation pathway in mammals, playing an important role in the detoxification and biotransformation of xenobiotics including mycotoxins such as deoxynivalenol (DON). Culmorin (CUL), a potentially co-occurring *Fusarium* metabolite, was recently found to inhibit the corresponding detoxification reaction in plants, namely DON-glucoside formation, raising the question whether CUL might affect also the mammalian counterpart. Using cell-free conditions, CUL when present equimolar (67 µM) or in fivefold excess, suppressed DON glucuronidation by human liver microsomes, reducing the formation of DON-15-glucuronide by 15 and 50%, and DON-3-glucuronide by 30 and 50%, respectively. Substantial inhibitory effects on DON glucuronidation up to 100% were found using the human recombinant uridine 5′-diphospho-glucuronosyltransferases (UGT) 2B4 and 2B7, applying a tenfold excess of CUL (100 µM). In addition, we observed the formation of a novel metabolite of CUL, CUL-11-glucuronide, identified for the first time in vitro as well as in vivo in piglet and human urine samples. Despite the observed potency of CUL to inhibit glucuronidation, no significant synergistic toxicity on cell viability was observed in combinations of CUL (0.1–100 µM) and DON (0.01–10 µM) in HT-29 and HepG2 cells, presumably reflecting the limited capacity of the tested cell lines for DON glucuronidation. However, in humans, glucuronidation is known to represent the main detoxification pathway for DON. The present results, including the identification of CUL-11-glucuronide in urine samples of piglets and humans, underline the necessity of further studies on the relevance of CUL as a potentially co-occurring modulator of DON toxicokinetics in vivo.

**Electronic supplementary material:**

The online version of this article (10.1007/s00204-019-02459-w) contains supplementary material, which is available to authorized users.

## Introduction

Mycotoxins are toxic compounds formed as secondary metabolites by different fungal genera such as *Fusarium, Aspergillus, Penicillium* and *Alternaria.* As toxigenic molds frequently contaminate agricultural crops pre- or post-harvest, mycotoxins may enter the food and feed chains, posing a potential risk to both human and animal health. Recent multi-mycotoxin surveys as well as human biomonitoring studies point at co-occurrence of various mycotoxins (Kovalsky et al. 2016; Marin et al. 2018; Warth et al. 2013b). It is rather the rule than the exception to be exposed simultaneously to a mixture of several mycotoxins through the diet. Nonetheless, mycotoxin risk assessment is still predominantly based on single-compound toxicity studies (EFSA et al. 2018, 2017a, b), and combinatory interactions, in particular with co-occurring fungal metabolites considered themselves to be of low toxicological relevance, are yet rarely taken into account.

Glucuronidation is a major phase II conjugation pathway for xenobiotics in most mammalian species. Glucuronide-conjugation of mycotoxins, including deoxynivalenol (DON), has been investigated in several studies (Maul et al. 2012; Schwartz-Zimmermann et al. 2017). Uridine 5′-diphospho-glucuronosyltransferases (UDP-glucuronosyltransferases, UGTs), integral membrane proteins localized in the endoplasmic reticulum, catalyze this transfer of glucuronic acid to the substrate (Dong et al. 2012). In humans, 22 UGT isoforms exist, which were classified into four gene families: UGT 1, UGT 2, UGT 3 and UGT 8 (Rowland et al. 2013). Regarding UGT tissue localization, numerous studies reported that in humans, the liver shows the highest abundance of UGT enzymes (Court et al. 2012; Izukawa et al. 2009; Ohno and Nakajin 2009), while extra-hepatic drug metabolism is considered to occur predominantly in kidneys and the gastrointestinal tract (Tourancheau et al. 2018; Tukey and Strassburg 2000).

**Fig. 1:**
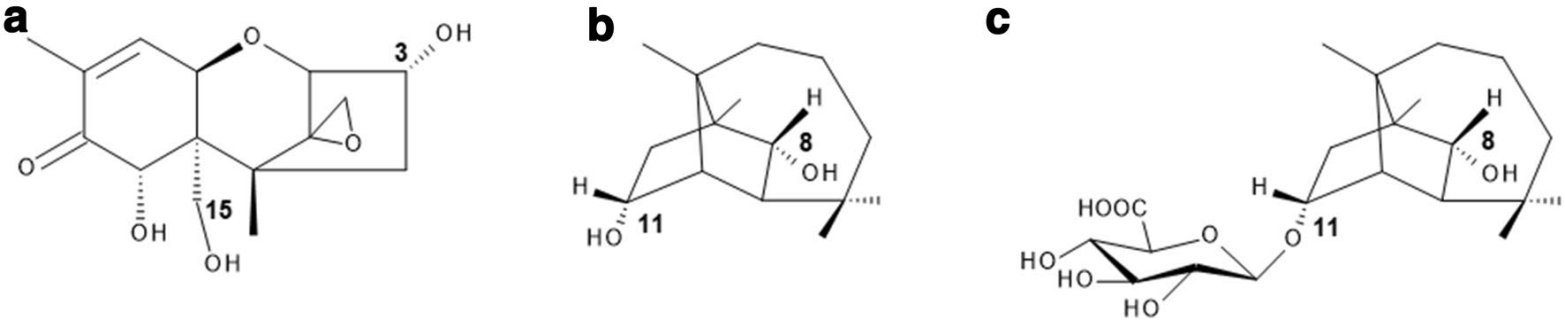
Chemical structures of the investigated *Fusarium* secondary metabolites and the newly identified glucuronidated CUL metabolite: (**a**) deoxynivalenol (DON), (**b**) culmorin (CUL), and (**c**) CUL-11-glucuronide (CUL-11-GlcA). Positions for potential glucuronidation are indicated

DON (Fig. 1a), a type-B trichothecene, is one of the most abundant *Fusarium* mycotoxins in temperate climate regions (EFSA et al. 2017b; Kovalsky et al. 2016; Streit et al. 2013). Due to its strong emetic effect, it is also referred to as vomitoxin. The C12–C13 epoxide moiety was shown to be crucial for its main mechanism of action, the interaction with the ribosomal 60S subunit, resulting in the inhibition of protein bio-synthesis (Garreau de Loubresse et al. 2014; Ueno 1977) and the induction of ribotoxic stress (Iordanov et al. 1997; Laskin et al. 2002; Pestka et al. 2004). Further mechanisms associated with DON exposure comprise, among others, pro-inflammatory processes (Pestka 2008, 2010a) and the activation of autophagic reactions (Del Favero et al. 2018). In several human biomonitoring studies, between 66 and 91% of ingested DON was excreted into urine as two different glucuronide conjugates: DON-3-glucuronide (DON-3-GlcA) and DON-15-glucuronide (DON-15-GlcA), the latter identified as the dominating isomer in humans (EFSA et al. 2017b; Vidal et al. 2018; Warth et al. 2013a). Maul et al. (2015) tested twelve commercially available human recombinant UGTs, whereof two isoforms led to the formation of the DON-glucuronides. UGT 2B4 predominantly formed DON-15-GlcA, whereas UGT 2B7 mainly catalyzed the formation of DON-3-GlcA.

The sesquiterpene diol culmorin (CUL, Fig. 1b), firstly isolated by Ashley et al. (1937), is another secondary metabolite produced by various *Fusarium* species, such as *F. culmorum, F. graminearum, F. venenatum* and *F. cerealis (*syn. *crookwellense*) (Greenhalgh et al. 1984; Lauren et al. 1987; Miller and MacKenzie 2000; Pedersen and Miller 1999). Occurrence levels of this fungal metabolite typically correlate with DON contamination levels, resulting in CUL concentrations about 2–3 times higher than those found for DON (Ghebremeskel and Langseth 2001; Uhlig et al. 2013). CUL was detected in 63% of 82 analyzed feed samples sourced in Europe, America and Australia at median and maximum concentrations of 195 and 44,616 µg/kg, respectively (Streit et al. 2013). Further studies identified CUL in 95–100% of Norwegian barley, oats and wheat samples, reaching median and maximum levels of 2000 and 31,500 µg/kg, respectively (Uhlig et al. 2013). CUL is often referred to as ‘fungal secondary metabolite’. However, it might also be considered as an ‘emerging mycotoxin’, defined by Vaclavikova et al. (2013) as mycotoxins which are ‘neither routinely determined, nor legislatively regulated; however, the evidence of their incidence is rapidly increasing’. Information on the toxicological relevance of CUL is limited. In the Ames test, mutagenic effects could be excluded (Pedersen and Miller 1999). CUL was shown to mediate weak antifungal activity (Strongman et al. 1987) and to be phytotoxic to wheat coleoptile tissue at concentrations between 0.1 and 1 µM (Wang and Miller 1988). Administration of CUL-contaminated feed to caterpillars and swine for 7 days (25 mg/kg) and 21 days (2 mg/kg) did not affect negatively the weight and performance of the animals (Dowd et al. 1989; Rotter et al. 1992). Nevertheless, in the same studies, potential combinatory interactions of CUL (10 mg/kg diet) with DON (25 mg/kg diet) were reported, leading to a significant weight reduction and an increased mortality of *Helio zea* larvae, in comparison to the effects caused by DON alone (Dowd et al. 1989). In pigs such interactions of CUL and DON could not be confirmed (Rotter et al. 1992). However, recently Michlmayr et al. (unpublished) identified CUL as an inhibitor of the UDP-glucosyltransferase Os79, resulting in a decreased DON-3-glucoside formation. Whereas mammalian enzymes use UDP-glucuronic acid as a substrate for conjugation reactions, the equivalent in plants is UDP-glucose, catalyzing, e.g., the transfer to the hydroxyl group at the C-3 position of DON (Poppenberger et al. 2003).

Based on the recent finding by Michlmayr et al. (unpublished) *in planta*, we aimed to address the question whether co-occurrence and presence of CUL affects also the major DON-detoxifying pathway in humans, namely glucuronidation. Therefore, we performed glucuronidation assays, using commercially available rat and human liver microscomes, as well as UGT Supersomes™. In these experiments, a new glucuronide metabolite of CUL could be identified, CUL-11-glucuronide (CUL-11-GlcA, Fig. 1c). Furthermore, the presence of CUL-11-GlcA was confirmed in urine samples of CUL-fed pigs and in a human urine sample. In addition, we determined the transcription levels of the UGTs relevant for DON glucuronidation (UGT 2B4, UGT 2B7) in human cell lines originating from intestine (HT-29, Caco-2) and liver (HepG2), and investigated whether inhibitory effects of co-occurring CUL might result in synergistic toxicity of DON and CUL.

## Materials and methods

### Chemicals and reagents

CUL was produced and purified as described in Weber et al. (2018). CUL-11-GlcA was produced from rat liver microsomes according to a similar protocol as used by Schwartz-Zimmermann et al. (2017). In total, 1.5-mg CUL was treated with pooled liver microsomes from male Sprague–Dawley rats (BioIVT, Brussels, Belgium), yielding one main substance with a glucuronidation yield of  > 95%. The product was isolated by preparative HPLC and consecutive NMR measurements revealed the compound to be CUL-11-GlcA. Details on NMR analysis and structure confirmation are presented in the Online Resource (Figs. S1, S2 and Table S1). DON was purchased from Romer Labs (Tulln, Austria). CUL was dissolved in dimethyl sulfoxide (DMSO) at 50 mM and DON in water (LC–MS grade) to obtain a stock solution of 10 mM. Recombinant human UGT 2B4 and UGT 2B7 were purchased from Corning Life Sciences (Amsterdam, Netherlands). Human liver microsomes (pooled from 25 donors of mixed gender, 20 mg/mL) were purchased from BioIVT (Brussels, Belgium). Alamethicin, magnesium chloride, uridine-diphosphoglucuronic acid (UDPGA) and uridine-diphospho-*N*-acetylglucosamine were obtained from Sigma Aldrich (Vienna, Austria). For mass spectrometric measurements, methanol (MeOH), acetonitrile (ACN), acetic acid and water of LC–MS grade were purchased from Sigma (Fluka; Vienna, Austria). A multi-component standard including DON and its metabolites (DON-3-sulfate, DON-15-sulfate, DON-3-GlcA and DOM-1) was prepared according to Warth et al. (2016). In addition, a second standard mix containing CUL and CUL-11-GlcA was prepared. For the animal experiment, polyethylene glycol 300 (PEG 300, Merck, Germany) was mixed with purified water (50/50, v/v). Thereafter, appropriate amounts of CUL were dissolved in 50% PEG 300 to obtain a stock solution of 300 µg/mL.

### Glucuronidation assay

The potential inhibitory effect of CUL on DON glucuronidation was analyzed using liver microsomes from rats or humans. In principle, the assays were performed as described previously for DON alone (Schwartz-Zimmermann et al. 2017). Three groups were set up: (1) DON (as positive control, *n* = 5), (2) CUL/DON (1:1 molar ratio, *n* = 5), (3) CUL/DON (1:5 molar ratio, *n* = 5). The concentration of DON in all cases was 20 mg/L (67 µM), while the molar excess of the co-substrate UDPGA was at least 20-fold. After stopping the reactions, samples were diluted 1:10 with 20% aqueous methanol and the formed glucuronides were determined by LC–MS/MS.

Furthermore, the effect of CUL on the glucuronidation of DON was tested using two different UGT isoforms (UGT 2B4, UGT 2B7) according to Maul et al. (2015). Human UGT Supersomes™ were used at a concentration of 1 mg/mL. Mixtures of the recombinant UGT isoform containing 100 mM potassium phosphate buffer (pH 7.4), 5 mM MgCl_2_ and 25 µg/mL alamethicin were placed on ice for 15 min to allow alamethicin pore formation. 100 µM CUL or the respective solvent control was added to the incubation solution and pre-incubated at 37 °C for 30 min. Afterwards, DON (10 µM) or CUL (100 µM) was added and the reaction was initiated by the addition of UDPGA (2.5 mM). After further incubation for 1 h at 37 °C, reactions were terminated by addition of 200 µL ACN. Samples were then chilled at − 20 °C for 10 min and centrifuged at 14,000×*g* for 5 min. Then, 200 µL of the supernatant were evaporated to dryness using a Labconco Centrivap Benchtop Vacuum Concentrator (Labconco, USA) and reconstituted in 100 µL water/ACN (9:1, v/v) for LC–MS/MS analysis. Glucuronidation experiments with the recombinant UGTs were carried out in four independent experiments.

### Animals and study design

All procedures related to the animal experiment were performed according to Austrian law and following the European Guidelines for the Care and Use of Animals for Research Purpose (European Commission 2010). The experiment was approved by the office of the Lower Austrian Region Government, Group of Agriculture and Forestry, Department of Agricultural Law (approval code LF1-TVG-39/050-2017) and carried out at the Center of Applied Animal Nutrition (Biomin Holding GmbH, Tulln, Austria).

Six crossbred piglets (sow: Landrace x Large White, boar: Pietrain; approximately 5 weeks old, 8.7 ± 1.0 kg, mixed sex) were obtained from a local swine producer. Piglets were housed individually in metabolic cages, had free access to water and were allowed to acclimatize for 5 days. Feed was provided twice daily and withdrawn after 30 min to monitor feed intake. Prior to the start of the experiment, the feed was tested for mycotoxin contamination by LC–MS/MS (Malachova et al. 2014). Low levels of CUL (20 µg/kg), 5-hydroxyculmorin (107 µg/kg), 15-hydroxyculmorin (67 µg/kg) and DON (79 µg/kg) were present in the diet, whereas other relevant mycotoxins (e.g., aflatoxin B_1_, fumonisin B_1_, ochratoxin A, etc.) were not detected.

Piglets received 50% PEG 300 (solvent control) and CUL (150 µg/kg body weight) by gavage on day 1 and 7. To this end, respective solutions were orally administered via polyvinyl chloride catheters. Urine and feces of individual piglets were collected 0–8 h, 8–24 h and 24–28 h after each of the treatments. Samples were stored at − 20 °C until analysis.

### LC–MS measurements

Pig urine samples were diluted to 0.2 mM creatinine with MeOH/water (50/50, v/v) and centrifuged at 14,350×*g* for 10 min. Measurement of liver microsome samples and pig urine samples was carried out on a 1290 Infinity series UHPLC system (Agilent Technologies, Waldbronn, Germany) coupled to a 6500+ QTrap mass spectrometer equipped with an IonDrive TurboV source (SCIEX, Foster City, CA, USA). Analytes were separated in gradient elution mode on a Kinetex C18 column (150 × 2.1 mm, 2.6 µm, Phenomenex, Aschaffenburg, Germany). Mobile phase A was water/acetic acid (99.9/0.1, v/v), mobile phase B was composed of ACN/acetic acid (99.9/0.1, v/v). The following linear gradient was used: 0.0–0.5 min: 5% B, 0.5–7.0 min: linear increase to 15% B, 7.0–14.0 min: linear increase to 90% B, 14.1–16.0 min: 100% B, 16.1–19.0 min: re-equilibration at 5% B. The flow rate was 250 µL/min, the column temperature was 30 °C and the injection volume was 3 µL. Mass spectrometric detection was performed in negative mode after electrospray ionization. The source parameters were: source temperature 400 °C, ion spray voltage − 4500 V, curtain gas 35 psi, ion source gas 1 60 psi and ion source gas 2 40 psi. Selected reaction monitoring mode was chosen as scan type and the following transitions were used: CUL-11-GlcAc quantifier *m/z* 413.5 → *m/z* 113.1 [declustering potential (DP) − 50 V, collision energy (CE) − 40 V], CUL-11-GlcAc qualifier_1 *m/z* 413.5 → *m/z* 175.1 (DP − 50 V, CE − 35 V), CUL-11-GlcAc qualifier_2 *m/z* 413.5 →  *m/z* 219.2 (DP − 50 V, CE − 50 V); CUL quantifier *m/z* 297.4 → *m/z* 59.0 (DP − 50 V, CE − 35 V). Analyst software version 1.6.3 (SCIEX) was used for instrument control and data analysis. CUL-11-GlcAc eluted at 11.40 min, CUL at 12.89 min. In urine diluted to 0.2 mM creatinine, matrix effects of CUL-11-GlcAc and CUL were 96 and 95%, respectively. The limits of detection (LODs, signal–noise ratio 3:1) for CUL-11-GlcAc and CUL in pure solvent standard solution were 0.1 ng/mL and 2 ng/mL, respectively. In urine diluted to 0.2 mM creatinine, the LOD of CUL-11-GlcAc was 0.8 ng/mL and the LOD of CUL was 2.5 ng/mL. Limits of quantification (signal to noise ratio 10:1) were by the factor 3.3 higher than LODs.

All samples obtained from the glucuronidation assay using the recombinant enzymes UGT 2B4 and UGT 2B7 and also the human urine sample were measured by LC–HRMS applying the following method: after reconstitution, the samples were analyzed by an LC–HRMS method using a UHPLC Vanquish system hyphenated with a Q Exactive HF high-resolution mass spectrometer equipped with a heated electrospray ionization (hESI) source (all from Thermo Fisher Scientific, Waltham, USA). Chromatographic separation was achieved on a reversed-phase Acquity UPLC HSS T3 column (100 × 2.1 mm, 1.8 μm, Waters, Milford, USA) protected by a HSS T3 VanGuard pre-column at 40 °C. At a constant flow rate of 0.4 mL/min, a linear gradient was employed with water (A) and ACN (B), both containing 0.1% acetic acid. After an initial hold time of 2 min at 10% B, the gradient was raised to 50% B at minute 6 and 100% B at minute 7. After 2 min at 100% B, the column was re-equilibrated at 10% B for 1 min. A sample volume of 2 μL was injected by the autosampler which was maintained at 4 °C. The following settings were used: negative ionization mode, capillary temperature, 325 °C; vaporizer temperature, 400 °C; sheath gas, 60 arbitrary units (a.u.); auxiliary gas, 20 a.u.; sweep gas, 3 a.u.; capillary voltage, 3 kV. The instrument was operated in profile mode (scan range, *m*/*z* 60–900) with a resolving power of 120.000 full width at half maximum and automatic gain control setting of 3 × 10^6^ with a maximum injection time of 100 ms. The LOD for DON-3-GlcA and DON-15-GlcA (peak area 5000 a.u.) was estimated based on the smallest peak detected within the respective measurement sequence. For data evaluation, Xcalibur™ (version 3.0, Thermo Scientific) and TraceFinder™ (version 3.3, Thermo Scientific) software were used. The general status of the instrument was regularly checked by the measurement of blank and quality control samples before and after the sequence.

The diluted human urine sample, obtained from pregnant women from Croatia (Sarkanj et al. 2013; Warth et al. 2016), was analyzed by the LC–HRMS full scan method to evaluate if CUL-11-GlcA may be found in human specimen. To verify the identity of the conjugate, additional MS/MS spectra of the urine sample and the reference standard were performed.

### Cell culture and treatment

The human colorectal adenocarcinoma cell line HT-29 was purchased from the German Collection of Microorganisms and Cell Cultures (DSMZ, Braunschweig, Germany), the human liver hepatocellular carcinoma cells HepG2 and the human colorectal adenocarcinoma cell line Caco-2 from the American Type Culture Collection (ATCC, Manassas, USA). For HT-29 and Caco-2 cell cultivation Dulbecco’s Modified Eagle’s Medium and for HepG2 cells RPMI 1640 medium was used. All three basal media formulations were supplemented with 10% (v/v) heat-inactivated fetal calf serum and 1% (v/v) penicillin/streptomycin (50 U/mL). Caco-2 culture medium was additionally supplemented with 1% potassium pyruvate and 0.01 mg/mL insulin–transferrin–selenium. Culture media and supplements were purchased from GIBCO Invitrogen (Karlsruhe, Germany). For cell cultivation and treatments, humidified incubators at 37 °C and 5% CO_2_ were used. Cells were routinely tested for the absence of mycoplasma contamination and used for experiments at passages 9–25. CUL, DON and their combinations were added to the incubation solutions, resulting in final solvent concentrations of 1% (v/v) LC–MS grade water and 1% (v/v) DMSO. Measurements of combinatory effects were always performed in parallel to the single substance on the same 96-well plate, to maximize comparability of the data and to allow an accurate calculation of the combinatory effects.

### Quantitative analysis of UGT gene transcription

Gene transcription of UGT 2B4 and UGT 2B7 in three different human cancer cell lines (HepG2, HT-29 and Caco-2) and the potential impact of CUL and DON on UGT transcription levels was analyzed by quantitative real-time PCR (qPCR). Cells were seeded in 24-well plates (HepG2: 52,500 cells/well; HT-29: 30,000 cells/well; Caco-2: 80,000 cells/well) and allowed to grow for 48 h. After respective incubations, total RNA was extracted using RNeasy^®^ Mini Kits (Qiagen, Hilden, Germany) and reversed transcribed into complementary DNA (cDNA) by QuantiTect^®^ Reverse Transcription Kit (Qiagen) according to the supplier’s protocols. Amplification of the cDNA samples in the presence of gene-specific primers (QuantiTect^®^ Primer Assays, Qiagen) and QuantiTect^®^ SYBR Green Master Mix (Qiagen) was performed in technical duplicates using a StepOnePlus™ System (Applied Biosystems, Foster City, USA). The following primer assays were used: β-actin (ACTB1, Hs_ACTB1_1_SG, QT00095431); glyceraldehyde 3-phosphate dehydrogenase (GAPDH, Hs_GAPDH_2_SG, QT01192646); UGT 2B4 (Hs_UGT2B4_1_SG; QT00029456); UGT 2B7 (Hs_UGT2B7_2_SG, QT01667554). A universal PCR protocol was applied including 15-min enzyme activation at 95 °C, 40 cycles of 15 s at 94 °C, 30 s at 55 °C and 30 s at 72 °C. StepOnePlus^®^ software (version 2.3, Applied Biosystems, USA) was used for fluorescence signal quantification and further data analysis. Of each tested sample, at least three independent experiments were performed. Presented transcript data were normalized to the mean of transcript levels of endogenous control genes (ACTB1, GAPDH) applying if possible and necessary the ΔΔCt-method (Schmittgen and Livak 2008). Respective 2^−ΔCt^ data are shown as Online Resource (Fig. S5a–c).

### Combinatory effects on cell viability—sulforhodamine B assay

To determine combinatory effects of CUL with DON on cell viability, the sulforhodamine B (SRB) assay according to Skehan et al. (1990) was applied. For this purpose, reactions containing different concentrations of CUL and the respective DON concentration at a constant substance ratio of 10:1 were incubated. In both cell lines, 24-h incubations were conducted; whereas in HT-29 combinations, additionally experiments applying 48-h toxin treatment were assessed. HT-29 and HepG2 cells were seeded into 96-well plates and allowed to grow for 72 h. Cells were incubated for 24 h or 48 h with CUL and DON (CUL 0.01–100 µM; DON 0.01–10 µM) and the respective combinations. Subsequently, the cells were rinsed with pre-warmed phosphate-buffered saline, fixed with 5% (v/v) trichloroacetic acid incubated at 4 °C for 30 min. Afterwards, cells were washed four times with water, plates were dried overnight at room temperature and then stained for 1 h using a solution of 0.4% (w/v) SRB in 1% (v/v) acetic acid. To remove the remaining staining solution, cells were washed twice with water and 1% (v/v) acetic acid solution. Then, plates were dried at room temperature in the dark. Finally, 10 mM Tris buffer (pH 10; 100 µL per well) was used to dissolve the dye and single wavelength absorbance was read at 570 nm using a Cytation 3 Imaging Multi Mode Reader (BioTek, Bad Friedrichshall, Germany). A solution of 1% (v/v) triton X-100 served as positive control. Cell-free blank values were subtracted and measured data were referred to the respective solvent control. Combinatory cell viability data were compared to single treatments and to a mathematically determined expected value (EV; see “Data visualization and statistical analysis of combinatory effects”).

### Data visualization and statistical analysis of combinatory effects

Combinatorial interactions of two or more substances are present if the experimentally determined effect differs from the “additive effect” of the two or more single compounds (Chou 2006). To assess interactions that do not follow a linear dose–response relationship, mathematical models must be applied to calculate this “additive effect”. Chou’s “multiple drug effect equation” (Chou 2006) is currently recognized as the most accurate model existing to describe these effects (Aichinger et al. 2018; Alassane-Kpembi et al. 2017; Vejdovszky et al. 2017). However, the determination of an IC_50_ value and, therefore, a measurable effect of at least 50% is a prerequisite for the application of this model. Since in some of our experimental setups this criterion was not fulfilled, the model of independent joint action (IJA) was chosen for the analysis of combinatory effects of CUL and its co-contaminant DON (Webb 1963). Applying this model, an EV is calculated for each combination taking into consideration the determined single effects via the formula: $f_{\text{ab}} = f_{\text{a}} + f_{\text{b}} - f_{\text{a}} f_{\text{b}}$, with $f_{\text{ab}}$ being the EV, and $f_{\text{a}}$ and $f_{\text{b}}$ being the measured effects of the single substances. Then, the EV was compared to the measured combinatory data set applying two-tailed Student’s *t* test. In the glucuronidation assay, statistical differences between the determined single effects of DON and the combination with CUL in glucuronide formation were calculated applying two-tailed Student’s *t* test using for the combinatory data 50% relative standard deviation (RSD), defined as LOD. For statistical analysis and data visualization, Origin 2018 software (Northampton, USA) was used.

## Results

### Inhibitory effect of CUL on in vitro glucuronidation of DON

**Fig. 2:**
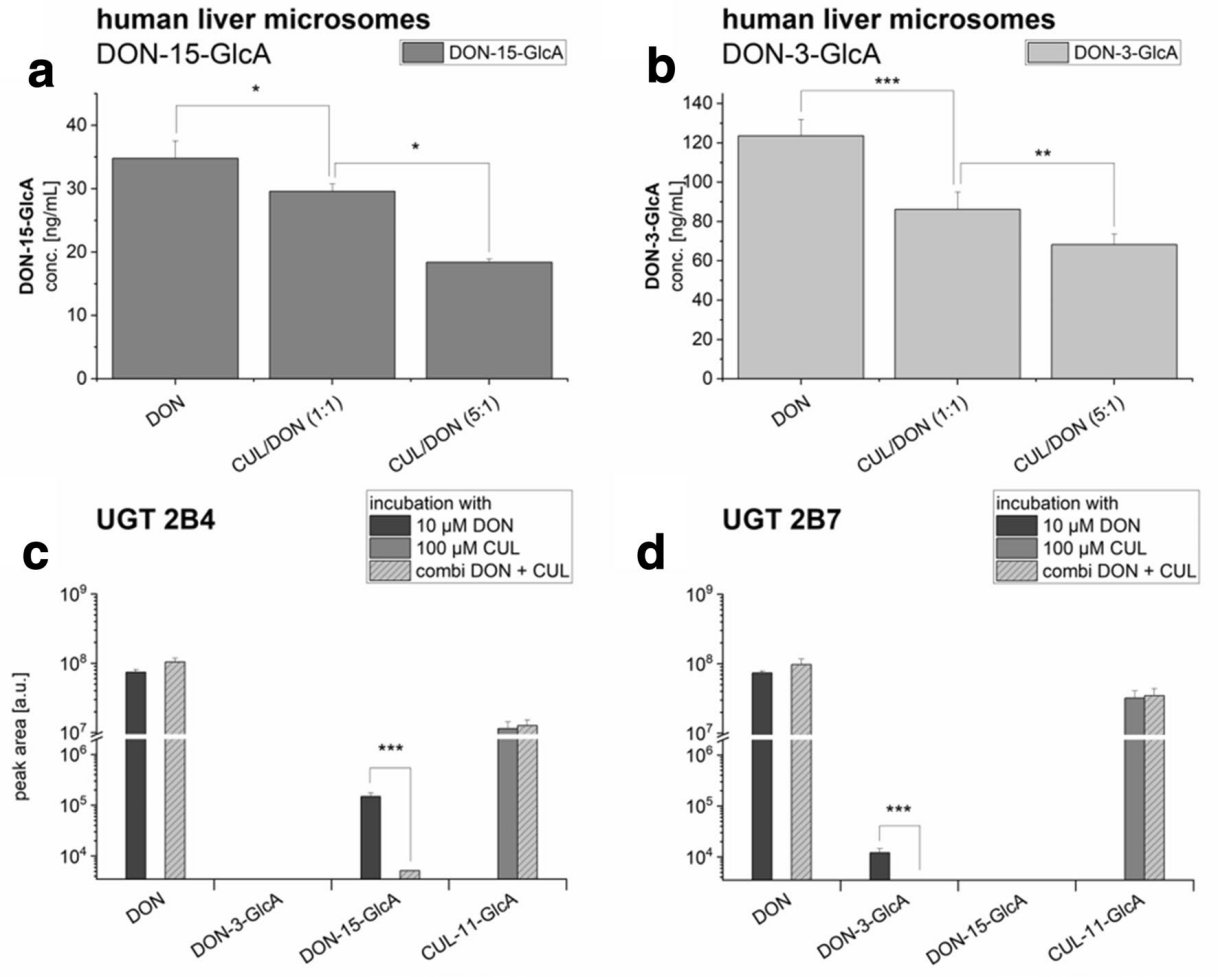
Glucuronidation activity of (**a** and **b**) human liver microsomes and of (**c**) the recombinant human UGT 2B4 and (**d**) UGT 2B7, mainly responsible for conjugation of DON and the respective combinatory effects with CUL. Data are expressed as mean values ± SD of four independent UGT incubations and of five independent microsomal incubations. Significant differences to the respective combinatory value are indicated with (*) representing *p *< 0.05, (**) representing *p *< 0.01 and (***) representing *p *< 0.001. For the statistical analysis of the recombinant UGTs 50% RSD, defined as LOD, were used

The ability of CUL to interfere with DON glucuronidation was assessed using two commercially available liver microsome preparations from human and rats. As expected, the main metabolite of DON incubated with liver microsomes from rats was DON-3-GlcA (data not shown). No significant concentration differences of DON-3-GlcA in the DON, CUL/DON (1:1) and CUL/DON (1:5) groups were seen. Thus, it seems that CUL has no modulatory effect on the glucuronidation activity of rat liver microsomes. Interestingly, the picture was quite different using human liver microsomes (Fig. 2a, b). Only a small part of DON was glucuronidated, of which the major metabolite was DON-15-GlcA (Fig. 2a), formed about 3.5 times more than DON-3-GlcA (Fig. 2b). However, using the human microsomes, combinatory incubations resulted in a significant decrease in the concentration of both formed DON-glucuronides with increasing CUL concentrations. In the equimolar combinations, only 85% DON-15-glucuronide and 70% DON-3-glucuronide of the initial glucuronide formation were found. Applying fivefold molar excess of CUL resulted in about 50% inhibition.

In case of human recombinant UGTs, both UGT 2B isoforms tested (2B4 and 2B7), accepted DON as a substrate, leading to the formation of one glucuronide each (Fig. 2c, d), even though the activities of both recombinant enzymes for the respective glucuronide formation were low. UGT 2B4 primarily formed DON-15-GlcA (Fig. 2c), whereas UGT 2B7 solely catalyzed the formation of DON-3-GlcA (Fig. 2d).

Combinatory incubations of 100 µM CUL together with 10 µM DON resulted in reduced glucuronidation activity in the enzyme preparations. In the UGT 2B4 incubations, the DON-15-GlcA signal was significantly reduced to 3% of the initial glucuronide formation, whereby signal detection was possible in only one of the four replicates very close to the method’s detection limit. In UGT 2B7, which showed lower glucuronidation activity with DON alone, the combination with CUL decreased glucuronidation to a level not detectable by the applied method.

When incubated with 100 µM CUL, both UGT 2B isoforms also led to the formation of a CUL-11-GlcA (Fig. 1c), firstly identified in in vitro enzyme incubations. CUL-11-GlcA formation was not affected by the presence of DON.

### Differences in UGT gene transcription in HepG2, HT-29 and Caco-2 cells

**Fig. 3:**
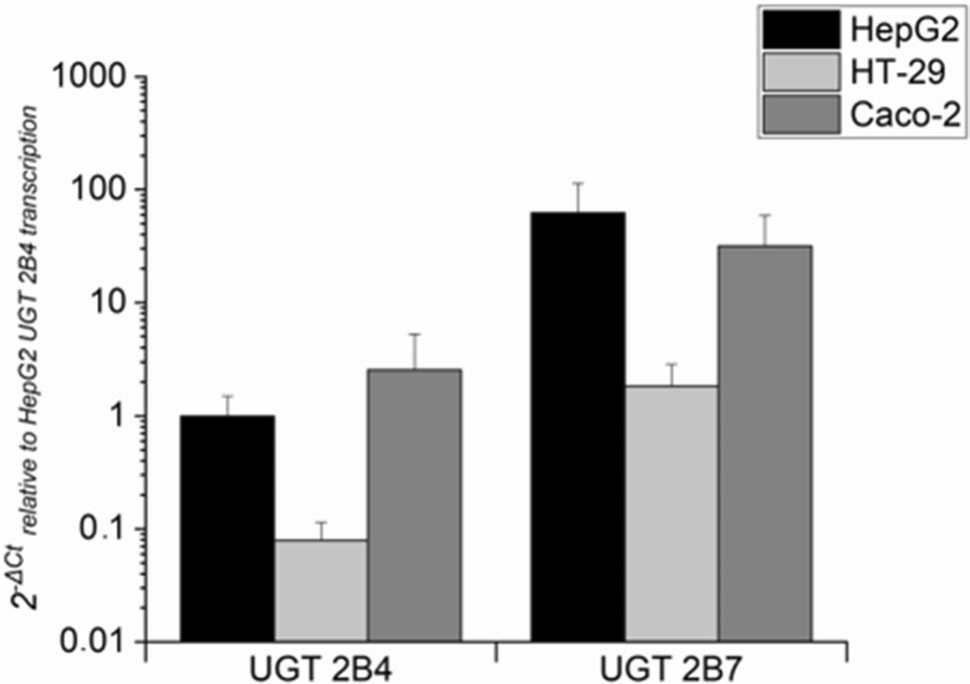
Gene transcription levels of UGT 2B4 and UGT 2B7 in HepG2, HT-29 and Caco-2 cells measured by qPCR. Transcription data are normalized to the gene expression levels of UGT 2B4 in HepG2. Data are expressed as mean values ± SD of at least four independent experiments performed in technical duplicates

Encouraged by these results, we set out to test whether an interaction between DON and CUL is also evident at the level of cell lines in toxicity assays. We first tested whether the relevant UGTs are expressed in the cell lines used, and whether the presence of DON or CUL had an effect on expression of the respective genes. Messenger RNA (mRNA) levels of the two UGT isoforms responsible for DON glucuronidation in three different cancer cell lines were investigated by qRT-PCR and are depicted relative to UGT 2B4 mRNA expression in HepG2 cells (Fig. 3). Transcription levels of UGT 2B4 in HepG2 and Caco-2 were found to be similar. In Caco-2, mRNA levels of UGT 2B4 were only 2.60 ± 2.67-fold higher than in HepG2. In contrast, in HT-29, mRNA of UGT 2B4 was found only at low levels in comparison to the other two cell lines, as a 0.07 ± 0.03-fold expression of this isoform was determined. Transcript levels of UGT 2B7 were much higher in the analyzed cell lines: about 62-fold in HepG2, only 1.8-fold in HT-29 and around 32-fold in Caco-2. Comparing the different UGT transcription levels of each cell line, in HepG2 cells, UGT 2B7 levels were comparably high. In HT-29, UGT 2B4 was determined to be the least transcribed isoform under the tested conditions. In Caco-2, a similar transcription pattern as in HepG2 cells was found.

### Impact of sub-toxic concentrations on UGT gene transcription

**Fig. 4:**
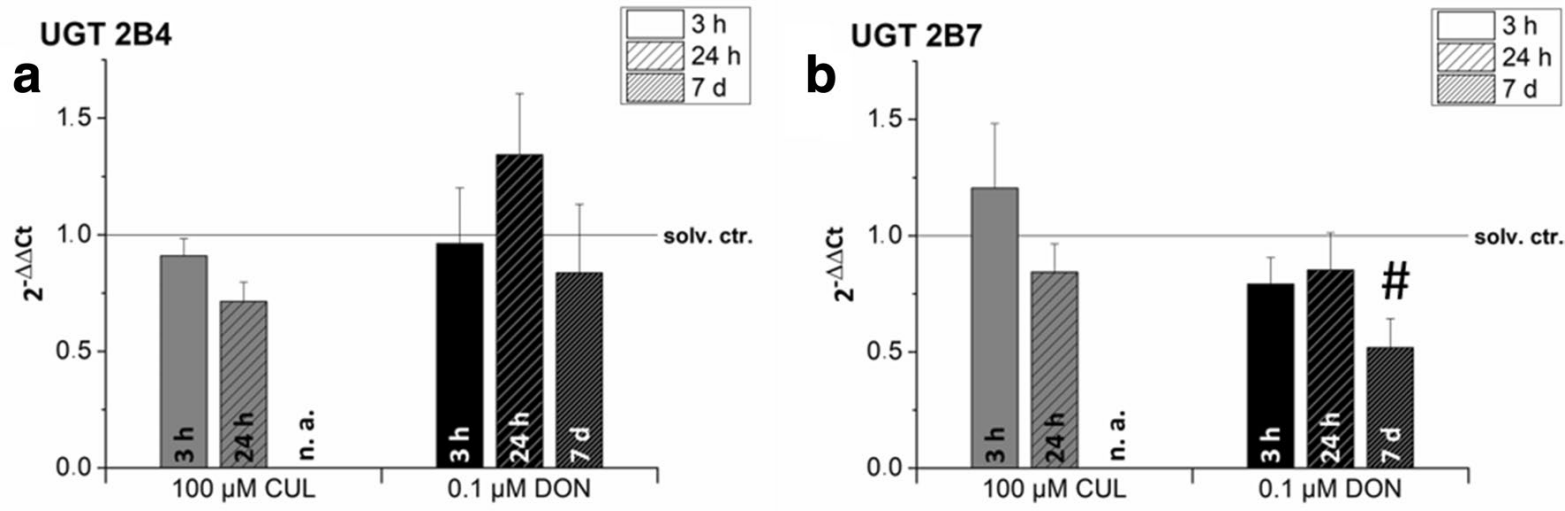
Impact of CUL and DON exposure on gene transcription levels of (**a**) UGT 2B4 and (**b**) UGT 2B7 in HT-29 cells after 3-h, 24-h and 7-day incubation measured by qPCR. Transcription data are normalized to the respective solvent controls (solv. ctr., solid line). Due to a lack of substance availability of CUL 7-day treatments have not been assessed in this case (n. a.). As sub-toxic substance concentrations were applied in this experiment, transcript levels are not affected by cytotoxicity. Data are expressed as mean values ± SD of at least three independent experiments performed in technical duplicates. Data were tested by Kruskal–Wallis ANOVA to compare different time-points and one-sample *t* test to assess differences to the solvent control. # indicates significant differences in comparison to the solvent control level (*p *< 0.05)

The human colon cancer cell line HT-29, known to effectively glucuronidate other mycotoxins such as alternariol and alternariol methyl ether (Pfeiffer et al. 2007), was selected exemplarily for further analysis of the UGT transcription levels and cytotoxicity studies (see “Combinatory cytotoxic effects”). qRT-PCR was used to investigate the modulatory effect of sub-toxic concentrations of CUL (100 µM) and DON (0.1 µM) after 3-h, 24-h and long-term exposure on UGT mRNA levels (Fig. 4a, b). Only marginal impact of short-term CUL exposure (3 h) on UGT transcription levels could be determined, resulting in a slightly increased mRNA concentration of UGT 2B7. However, 24-h incubation with 100 µM CUL resulted in a decrease in the transcription levels of both UGT isoforms tested. Due to limited availability (in-house purification of CUL), long-term cell exposure for 7 days with CUL was not tested.

Incubations with DON for 3 h caused a tentative reduction of the UGT 2B7 transcription level (Fig. 4b). While after 24-h cell exposure, UGT 2B4 transcript levels were increased to 1.3 ± 0.2, UGT 2B7 mRNA levels remained below solvent control level. Long-term treatment of HT-29 cells for 7 days caused a significant reduction of the transcription level in case of UGT 2B7 in comparison to the solvent control, resulting in the following relative transcript level: 0.5 ± 0.1 (Fig. 4b).

### Combinatory cytotoxic effects

**Fig. 5:**
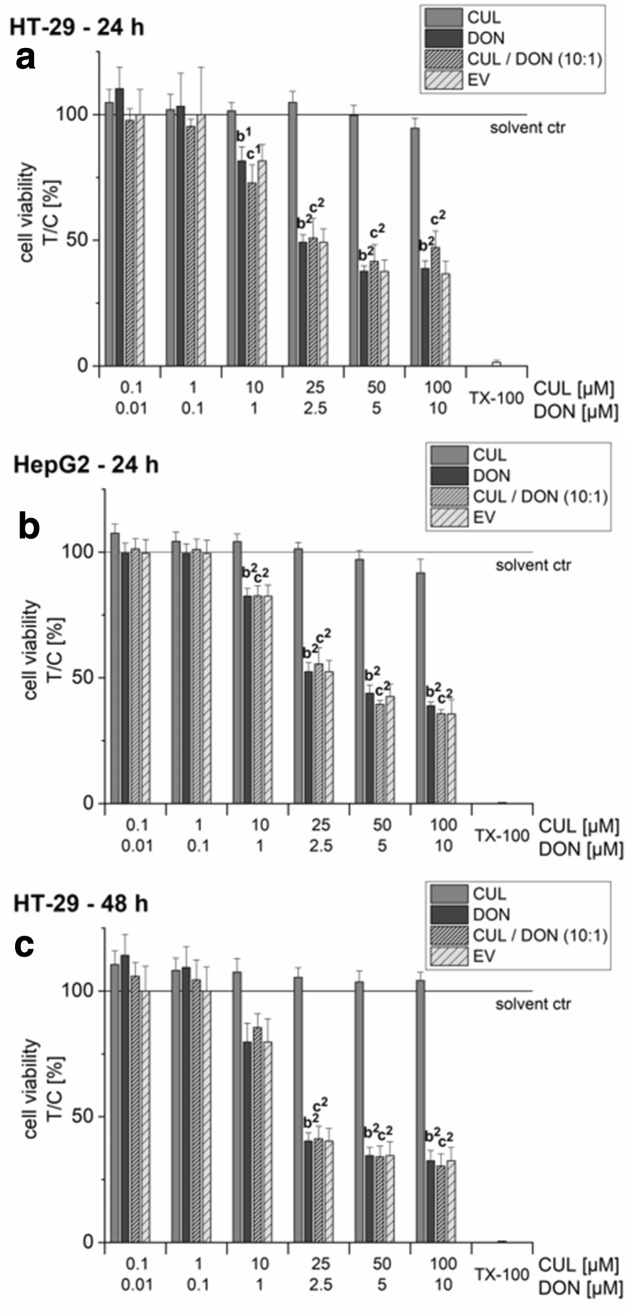
Combinatory effects of CUL with DON on cell viability of HT-29 (24-h incubation: **a**; 48-h incubation: **c**) and HepG2 cells (24-h incubation: **b**) in the sulforhodamine B assay. Combinations of CUL and DON were combined 10:1. 1% water (LC–MS grade) + 0.5% DMSO served as solvent controls (solid line). Data are expressed as mean values ± SD of at least five independent experiments performed in triplicates normalized to the respective solvent control. 1% triton-X 100 was used as positive control (TX-100). Significant differences to the respective lowest tested concentration are indicated in the graphs with (**b**) for DON and (**c**) for the combination (exponents represent: (1) *p* < 0.05 and (2) *p* < 0.01)

In both cell lines, HT-29 and HepG2, CUL (0.1–100 µM) did not cause any statistically significant cytotoxic effect in the SRB assay after 24-h incubation (Fig. 5a, b). Since a constant 10:1 ratio has already been used in the glucuronidation assay (see “Inhibitory effect of CUL on in vitro glucuronidation of DON”) and as DON concentrations ≥ 10 µM are known to cause strong cytotoxic effects after 24-h incubations, combinations with CUL were performed in the concentration ratio 10:1 (CUL:DON; Fig. 5a–c). In both cell lines, DON mediated similar cytotoxic effects, resulting in a statistically significant reduction of the cell viability at DON concentrations ≥ 1 µM. Combinations of CUL and DON caused similar cytotoxic effects to the ones mediated by DON alone and none of the tested combinations differed significantly from the mathematically calculated EVs.

To assess combinatory effects of CUL on DON-induced cytotoxicity after longer incubation times, HT-29 cells were treated for 48 h. Nevertheless, even cell treatments for 48 h did not cause significant cytotoxic effects of CUL, nor combinatory effects of CUL and DON (Fig. 5c). Thus, with respect to cytotoxicity, no significant effects of the binary mixtures in comparison to the single treatments with DON or the calculated EV were observed.

### Formation of CUL-11-GlcA in vivo

**Fig. 6:**
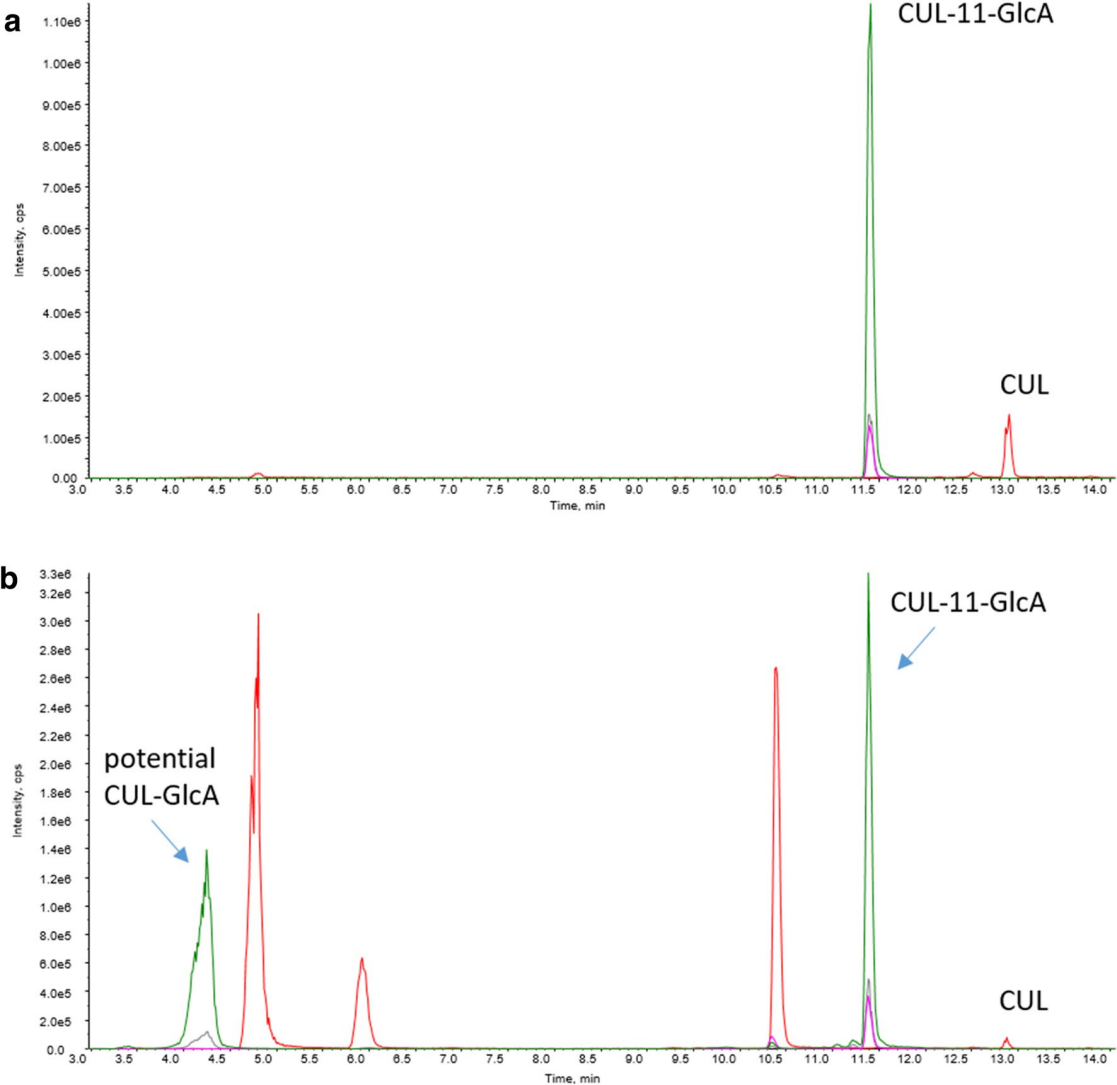
LC–MS/MS chromatograms of CUL-11-GlcA and CUL (**a**) in pure solvent standard solution (100 ng/mL of both compounds) and (**b**) in a piglet urine sample (concentration of CUL-11-GlcA in urine: 5.38 mg/L, concentration of CUL in urine: 0.71 mg/L)

Urine samples of the piglet trial were diluted to the same concentration of creatinine (0.2 mM) and concentrations of CUL and CUL-11-GlcA were determined by LC–MS/MS. CUL-11-GlcA eluted at 11.40 min, CUL at 12.89 min and a potential further CUL-GlcA at 4.27 min. In urine diluted to 0.2 mM creatinine, matrix effects of CUL-11-GlcA and CUL were 96 and 95%, respectively. The LODs (signal to noise ratio 3:1) for CUL-11-GlcA and CUL in pure solvent standard solution were 0.1 ng/mL and 2 ng/mL, respectively. In urine diluted to 0.2 mM creatinine, the LOD of CUL-11-GlcA was 0.8 ng/mL and the LOD of CUL was 2.5 ng/mL. Limits of quantification (signal–noise ratio 10:1) were by the factor 3.3 higher than LODs. Figure 6 shows chromatograms of CUL-11-GlcA and CUL in pure solvents (Fig. 6a) and in a pig urine sample (Fig. 6b).

As with the recombinant UGT enzymes and (human and rat) liver microsomes, CUL was also readily glucuronidated in vivo to CUL-11-GlcA by pigs. In measured urine samples from four animals, the concentration of CUL-11-GlcA was higher than of CUL (maximum CUL-11-GlcA/CUL ratio: 8.7, average ratio 6.3). In urine samples of two animals, no CUL-11-GlcA was detected. One likely explanation for the latter is that insufficient separation of urine and feces in the metabolic cages led to hydrolysis of the formed glucuronides by gut microbes during the sampling period.

CUL-11-GlcA was further detected in a human urine sample obtained from pregnant women in Croatia (Sarkanj et al. 2013; Warth et al. 2016) (Online Resource: Fig. S4). Retention time and MS/MS spectra (Fig. 6, Online Resource: Fig. S3 and S4) allowed the identification of CUL-11-GlcA as novel pig and human metabolite.

## Discussion

Mycotoxins not only cause substantial economic losses due to contaminated food, feed and loss of animal productivity, but also pose a serious health threat to both humans and livestock. Numerous studies tried to characterize the toxic potential and mechanistic activities of various *Fusarium* mycotoxins (Alshannaq and Yu 2017; Fraeyman et al. 2017; Pestka 2010b). Nevertheless, so far this study is, to the best of our knowledge, the first to address combinatory effects of the two co-occurring *Fusarium* metabolites DON and CUL in human cell models. CUL, previously reported to have limited toxic potential in mammals (Dowd et al. 1989; Miller and MacKenzie 2000; Pedersen and Miller 1999; Prelusky et al. 1989; Rotter et al. 1992), was found to inhibit the metabolic activity of purified plant UDP-glucosyltransferases (Michlmayr et al., unpublished). To investigate interactions of CUL and DON on DON-detoxifying, mammalian enzymes, glucuronidation activities of human liver microsomes and two human UGTs were determined. Using human liver microsomes, significantly lower levels of DON-glucuronides were found in the presence of CUL in a concentration-dependent manner (Fig. 2a, b). Regarding the two tested UGT isoforms, UGT 2B4 and 2B7, combinations with CUL led in both cases to conjugate concentrations near or even below the method’s detection limit (Fig. 2c, d). Furthermore, we confirmed that DON-15-GlcA is predominantly formed by UGT 2B4 and that DON-3-GlcA formation is mainly catalyzed by UGT 2B7 (Maul et al. 2015). In accordance with Maul et al. (2015), only low glucuronidation activities were observed by both UGTs.

In the course of the in vitro enzyme experiments, a new CUL metabolite, CUL-11-GlcA, was identified and characterized. This finding argues presumably for a competitive inhibition of the UGTs, as the terpenoid CUL and DON might compete for the same binding site on the enzyme. However, CUL-11-GlcA was not only detected after incubation with UGT 2B4 and 2B7 in vitro (Fig. 2), but also identified as a novel in vivo metabolite in pig urine samples and in a human urine sample obtained from pregnant women in Croatia (Fig. 6; Online Resource: Fig. S4) (Sarkanj et al. 2013; Warth et al. 2016). This sample has been demonstrated before to contain high concentrations of DON (275 µg/L) and of its metabolites DON-3-GlcA (298 µg/L), DON-15-GlcA (1238 µg/L), and DON-3-sulfate (58 µg/L) (Sarkanj et al. 2013; Warth et al. 2016). Furthermore, a second CUL-GlcA was detected in the pig urine sample, which due to a lack of reference standard could be only tentatively identified as CUL-8-GlcA (Fig. 6).

In cell culture experiments, we aimed to further evaluate potential synergistic toxic effects of DON and CUL probably resulting from the UGT inhibition. To select in this respect appropriate cell lines, expressing the two UGT isoforms of interest, three well-known cancer cell lines (HepG2, HT-29 and Caco-2) were tested for their UGT transcription levels (Fig. 3). Even though mRNAs of both isoforms were present in all the addressed cell lines, expression levels differed substantially when comparing the three cell models, but also among the different isoforms. So far, UGT mRNA expression levels of these cell lines have not been compared, even though numerous studies investigated already UGT expression levels in human tissues, focusing mainly on the liver and the gastrointestinal tract (Court et al. 2012; Izukawa et al. 2009; Ohno and Nakajin 2009; Strassburg et al. 2000; Tukey and Strassburg 2000; Wu et al. 2011). As all three selected cell lines are commonly used for various toxicological studies and also to investigate xenobiotic and drug metabolism in vitro (Akbari et al. 2017; Bohets et al. 2001; Weaver et al. 2017), this characterization of UGT transcription levels is of crucial importance for the scientific community. A study, using real-time PCR to generate a quantitative expression level profile of the UGTs in 26 adult and 3 fetal tissues, confirmed that liver is the organ containing the highest levels of UGT mRNAs, among which UGT 2B4 was the most abundant isoform (40% of total UGT mRNA content; up to 90% in fetal liver) (Court et al. 2012). Ohno and Nakajin (2009) analyzed beyond liver tissue extrahepatic mRNA levels of various UGT isoforms. Intestinal tissue expressed predominantly UGT 1A1, 1A10, 2B7, 2B15 and 2B17. Relatively low levels of various UGTs were reported in esophageal tissue and even in steroidogenic tissues, such as breast, prostate, heart and adrenal gland (Ohno and Nakajin 2009). However, most transcript profiles of UGTs in human tissue-derived cell lines are not consistent with those in corresponding tissue samples (Hart et al. 2010; Nakamura et al. 2008). Using semiquantitative reverse-transcription PCR Nakamura et al. (2008) determined UGT mRNA levels from various cell lines including HepG2 and Caco-2. In HepG2, all UGT 2B isoforms tested were all highly expressed, whereas comparably low mRNA levels of most 1A isoforms were determined. For Caco-2, similar UGT expression levels for UGT 1A1, 2B7 and 2B15 were elucidated; however, UGT 2B isoforms were less present in comparison to the levels in HepG2 cells (Nakamura et al. 2008). Studies comparing transcript levels or enzyme activities of HepG2 cells with those of human tissue samples, of primary human hepatocytes or of other liver cell lines, such as HepaRG, mainly reported in HepG2 cells lower UGT transcript levels and enzyme activities (Hart et al. 2010; Westerink and Schoonen 2007; Yokoyama et al. 2018). Performing a similar data analysis as Ohno and Nakajin (2009), normalizing the Ct-values obtained in our qRT-PCR experiments to the mean of transcript levels of endogenous control genes, multiplying by a factor 10^4^, relative values for UGT 2B4 of 5375 and 5026 and for UGT 2B7 of 6695 and 5781 can be obtained for HepG2 and HT-29, respectively. As Ohno and Nakajin (2009) reported in liver tissue samples values above 37,900 for UGT 2B4 expression, our results clearly show that the transcript levels of this UGT isoform in HepG2 cells are much lower than in the liver tissue. However, with respect to UGT 2B7, similar relative expression values to the ones calculated and reported above have been described by Ohno and Nakajin (2009).

Various studies in animal and in vitro models have demonstrated that the consumption of certain xenobiotics (e.g., aflatoxin B_1_, β-naphthoflavone, ethanol,etc.) may increase UGT levels and respective glucuronidation activities via different induction pathways (Court 2010; Hanioka et al. 2012, 2006; Kardon et al. 2000; Li et al. 2000). Since mRNA levels of the selected UGT isoforms were partly expressed at relatively low levels, exemplarily HT-29 cells were exposed to sub-toxic concentrations of DON (0.1 µM) and CUL (100 µM). We speculated that these short- and long-term incubations using respective UGT enzyme substrates might have modulatory or even inducing effects on UGT transcript levels (Fig. 4a, b). Incubations for 3 h only marginally affected UGT mRNA levels and after 24-h, UGT 2B4 was the only one slightly induced after DON incubation. Up to 2.5-fold inductions, such as reported by Hanioka et al. (2012) in HepG2 cells after 48-h aflatoxin B_1_ treatment, could not be corroborated in this study. To date, similar studies assessing modulatory effects of DON or CUL treatment on UGT mRNA levels have not been described in literature so far.

Despite the fact that UGT transcription levels in all three cell lines were relatively low and even an extended exposure to sub-toxic substrate concentrations could not induce UGT mRNA expression, potential synergistic effects of the two *Fusarium* secondary metabolites on cell viability were assessed (Fig. 5). In HT-29 and HepG2 cells after 24 h and 48 h, no significant interactions between CUL and DON could be determined. Hence, interactions between DON and CUL observed *in planta* (Michlmayr et al., unpublished) and by Dowd et al. (1989) in caterpillars, could not be confirmed in our cell models. While humans excrete in urine up to 90% of total DON as glucuronide conjugates, LC–MS/MS analysis of cell extracts could not detect DON-glucuronides in HT-29 and HepG2 cells (data not shown). However, in the same experiments, high glucuronide formation by both cell lines was observed for the *Alternaria* mycotoxin alternariol, known to be predominantly conjugated by UGT 1A1 and UGT 1A9 (data not shown). Thus, as also transcription levels of UGT 2B4 were about tenfold lower than levels reported in liver tissue (Ohno and Nakajin 2009), potential inhibitory effects of CUL might be of minor importance for the overall toxicity in cell culture and might not be measurable at the determined endpoints. However, negative evidence from our cell culture experiments does not exclude a synergistic effect in the mammalian organism. Due to a lack in exposure and toxicokinetic data, one can only speculate, if relevant systemic concentrations of CUL and DON, at which competitive inhibition of UGTs might take place, can be reached by dietary toxin exposure. As technical issues rendered the findings of the above-mentioned in vivo study inconclusive, a respective animal study needs to be repeated to gain further insights into these CUL–DON interactions.

In conclusion, the present study reported for the first time CUL to partially inhibit the glucuronidation activity of human liver microsomes and recombinant UGTs. Furthermore, we were able to detect two novel CUL-glucuronides both in vitro, as well as, in vivo in pig and human urine samples. Even though in HT-29 and HepG2 cells this presumably competitive effect of CUL on DON glucuronidation did not induce considerably higher cytotoxicity in mixtures, which may be due to the limited glucuronide formation in the tested cell lines, in the human organism DON-glucuronide formation is the main detoxification reaction. Hence, to elucidate if this inhibitory mechanism and resulting combinatory effects are of importance in the mammalian organism and to gain further insight into systemic CUL concentrations and respective toxicokinetics, a more detailed characterization in vivo is required. This may contribute substantially to the discussion about classifying CUL not only as a secondary fungal metabolite but also as an “emerging mycotoxin”. Furthermore, this study shows that we are still at the very beginning in the toxicological profiling of chemical mixtures, and underlines also that the assessment of drug-exposome interactions and their further understanding are of crucial importance.

## Electronic supplementary material

Below is the link to the electronic supplementary material.

Supplementary material 1 (PDF 710 kb)

## Abbreviations

ACN: Acetonitrile
ACTB1: Β-Actin
a.u.: Arbitrary unit
cDNA: Complementary deoxyribonucleic acid
CE: Collision energy
CUL: Culmorin
CUL-11-GlcA: Culmorin-11-glucuronide
CUL-GlcA: Culmorin-glucuronide
DP: Declustering potential
DMSO: Dimethyl sulfoxide
DON: Deoxynivalenol
DON-3-GlcA: Deoxynivalenol-3-glucuronide
DON-7-GlcA: Deoxynivalenol-7-glucuronide
DON-15-GlcA: Deoxynivalenol-15-glucuronide
EFSA: European food safety authority
EV: Expected value
GAPDH: Glyceraldehyde-3-phosphate dehydrogenase
LOD: Limit of detection
MeOH: Methanol
mRNA: Messenger ribonucleic acid
n. a.: Not assessed
PEG 300: Polyethylene glycol 300
qPCR: Quantitative polymerase chain reaction
RNA: Ribonucleic acid
RSD: Relative standard deviation
UDPGA: Uridine diphosphate glucuronic acid
UGT: Uridine 5′-diphospho-glucuronosyltransferase
