# Supplementary material for: The Fusarium metabolite culmorin suppresses the in vitro glucuronidation of deoxynivalenol
Source: Arch Toxicol. 2019 May 2;93(6):1729–43. doi: 10.1007/s00204-019-02459-w (PMC6620244; doi:10.1007/s00204-019-02459-w)
Supplement: Supplementary file 1 — Supplementary material 1 (PDF 710 kb) [file 204_2019_2459_MOESM1_ESM.pdf]

The *Fusarium* metabolite culmorin suppresses the *in vitro* glucuronidation of deoxynivalenol

Lydia Woelflingseder[a], Benedikt Warth[a,] Immina Vierheilig[a], Heidi Schwartz-Zimmermann[b], Christian Hametner[c], Veronika Nagl[d], Barbara Novak[d], Bojan Šarkanj[e], Franz Berthiller[b], Gerhard Adam[f], Doris Marko[a, *]

[a] Department of Food Chemistry and Toxicology, Faculty of Chemistry, University of Vienna, Währingerstrasse 38, 1090 Vienna, Austria: lydia.woelflingseder@univie.ac.at; immina.vierheilig@gmail.com; benedikt.warth@univie.ac.at; doris.marko@univie.ac.at.

[b] Christian Doppler Laboratory for Mycotoxin Metabolism and Center for Analytical Chemistry, Department of Agrobiotechnology (IFA-Tulln), University of Natural Resources and Life Sciences, Vienna (BOKU), Konrad-Lorenz-Strasse 20, 3430 Tulln, Austria: heidi.schwartz@boku.ac.at; franz.berthiller@boku.ac.at.

[c] Institute of Applied Synthetic Chemistry, Vienna University of Technology, Getreidemarkt 9/163, 1060 Vienna, Austria: christian.hametner@tuwien.ac.at.

[d] BIOMIN Research Center, Technopark 1, 3430 Tulln, Austria: veronika.nagl@biomin.net, barbara.novak@biomin.net

[e] Department of Applied Chemistry and Ecology, Faculty of Food Technology, Josip Juraj Strossmayer University of Osijek, Franje Kuhača 20, 31000, Osijek, Croatia: bsarkanj@ptfos.hr

[f] Department of Applied Genetics and Cell Biology, University of Natural Resources and Life Sciences, Vienna (BOKU), Konrad-Lorenz-Strasse 24, 3430 Tulln, Austria: gerhard.adam@boku.ac.at.

* Corresponding author:

Univ.-Prof. Dr. Doris Marko: Department of Food Chemistry and Toxicology, Währingerstrasse 38, 1090 Vienna, Austria; Tel.: +43-1-4277-70800; doris.marko@univie.ac.at

CUL-11-GlcA: structure confirmation by nuclear magnetic resonance (NMR)

NMR spectra were obtained in acetone-d6 at 295 K on a Bruker Avance III HD spectrometer (Bruker BioSpin GmbH, Rheinstetten, Germany) equipped with a 5 mm Cryoprobe™ Prodigy BBO, operating at 600.15 MHz for ^{1}H and 150.90 MHz for ^{13}C. NMR data were recorded and evaluated using TopSpin 3.2 (Bruker BioSpin GmbH). All pulse programs were taken from the Bruker software library. Chemical shifts were established based on residual solvent signals (2.05 ppm for ^{1}H and 29.92 ppm for ^{13}C) and reported relative to tetramethylsilane (TMS).

Using 1D (^{1}H, ^{13}C-CPD) and 2D (^{1}H^{1}H-COSY, ^{1}H^{13}C-HSQC, and ^{1}H^{13}C-HMBC) NMR experiments, the structure was confirmed and all signals could be assigned. Specifically, the carbohydrate moiety was proven to be attached to position 11 of the CUL core by HMBC correlations between H-11 and C-1' as well as H-1' and C-11.

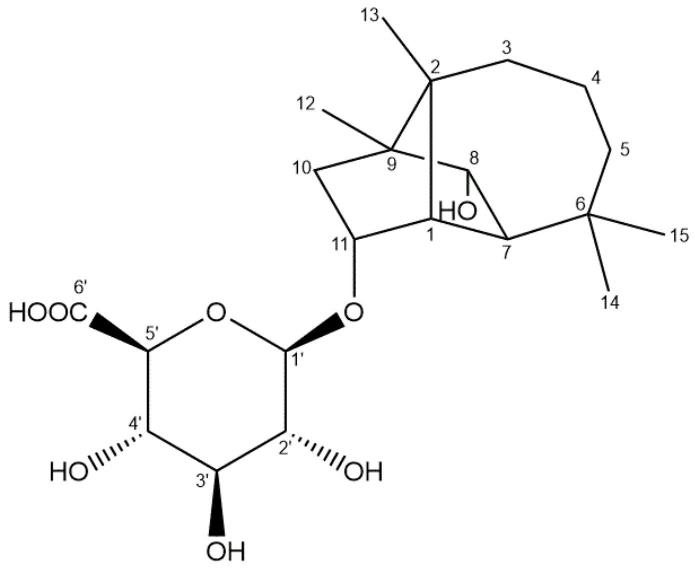

Fig. S1 ***Structure of CUL-11-GlcA and numbering scheme***

Table S1 *^{1}H and ^{13}C NMR data*

Pos.	^{1}H<br>δ [ppm] (multiplicity; J [Hz])	^{13}C<br>δ [ppm]
1	2.08 (m)	49.7
2	-	51.4
3	1.46 (m)<br>1.29 (m)	36.7
4	1.50-1.40 (m)	23.3
5	1.35-1.30 (m)	42.4
6	-	33.7
7	1.96 (m)	52.7
8	3.77 (dd; 5.1, 1.3)	79.2
9	-	52.7
10	1.95 (m)<br>1.55 (ddd; 13.5, 9.7, 2.0)	34.6
11	4.32 (dt; 9.8, 4.2)	80.0
12	0.78 (s)	13.6
13	0.87 (s)	22.7
14, 15	0.89 (s), 0.94 (s)	29.3, 29.9
1'	4.42 (d; 7.7)	104.2
2'	3.25 (dd; 9.1, 7.8)	74.8
3'	3.44 (t; 9.0)	77.1
4'	3.61 (dd; 9.0, 9.6)	72.8
5'	3.80 (d; 9.8)	76.3
6'	-	170.6

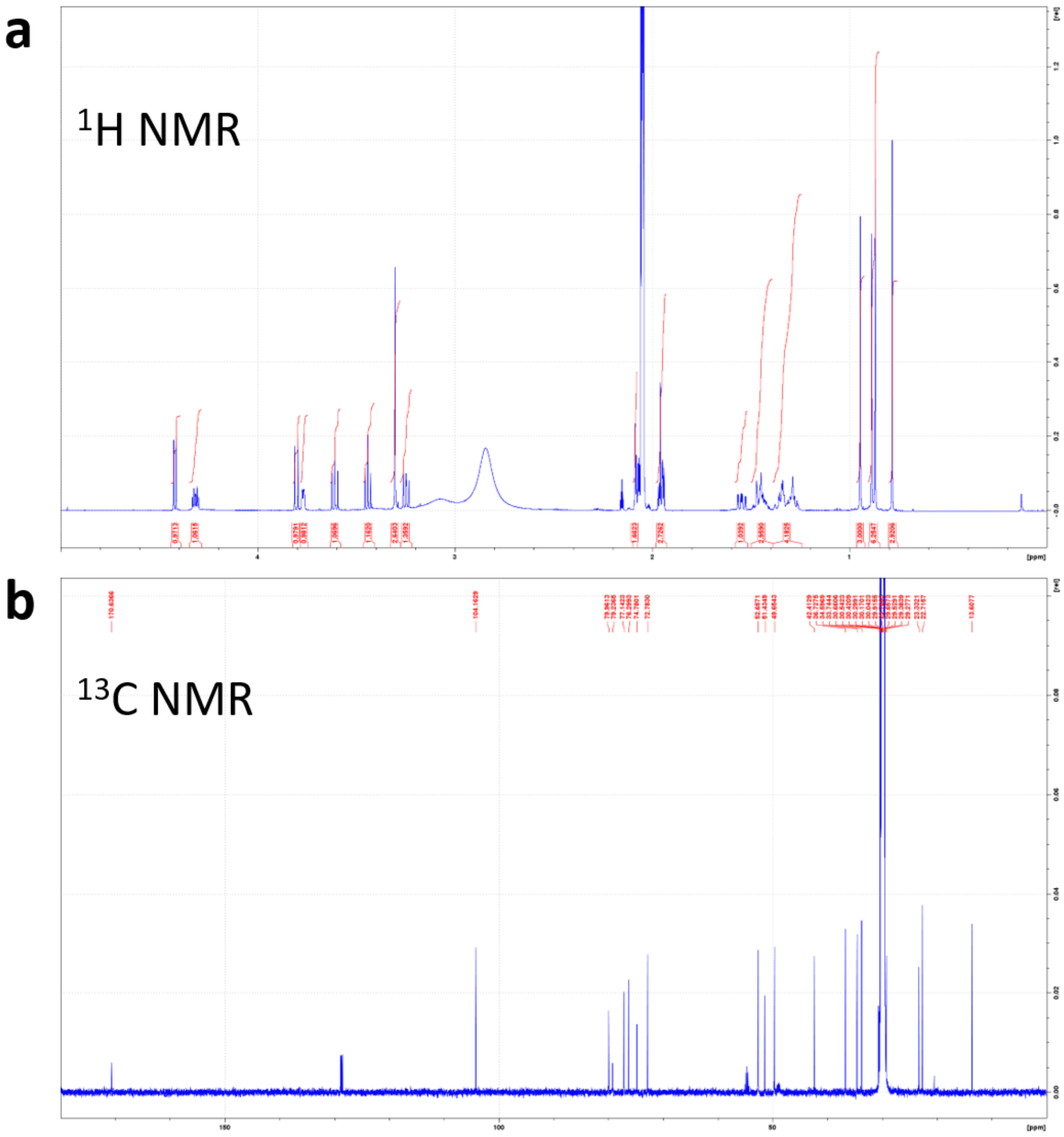

Fig. S2 ***(a)*** ***^{1}H NMR and (b) ^{13}C NMR spectra of CUL-11-GlcA***

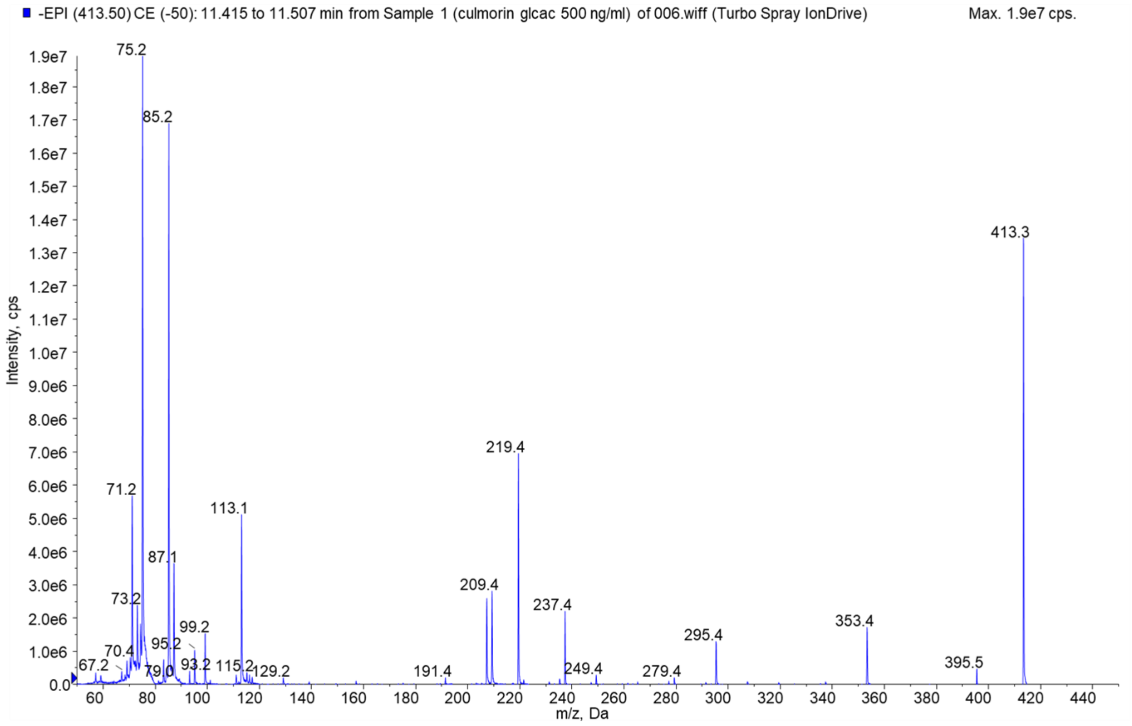

Fig. S3 ***MS/MS spectrum of CUL-11-GlcA (11.4 min) at a collision energy (CE) of -50 eV.***

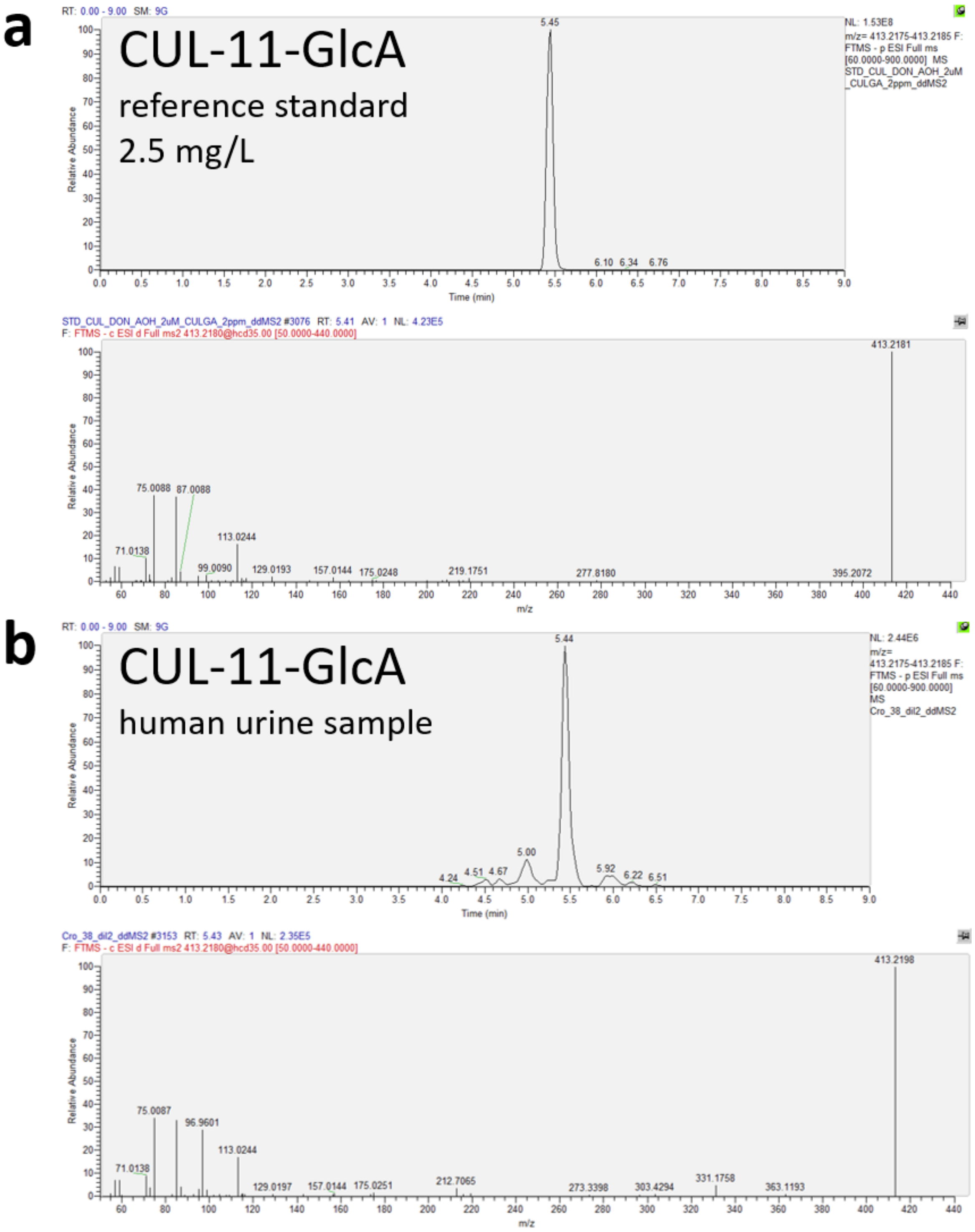

Fig. S4 ***Chromatogram and respective MS/MS spectra of CUL-11-GlcA in (a) a reference standard sample (2.5 mg/L) and (b) a diluted (1:10) human urine sample.***

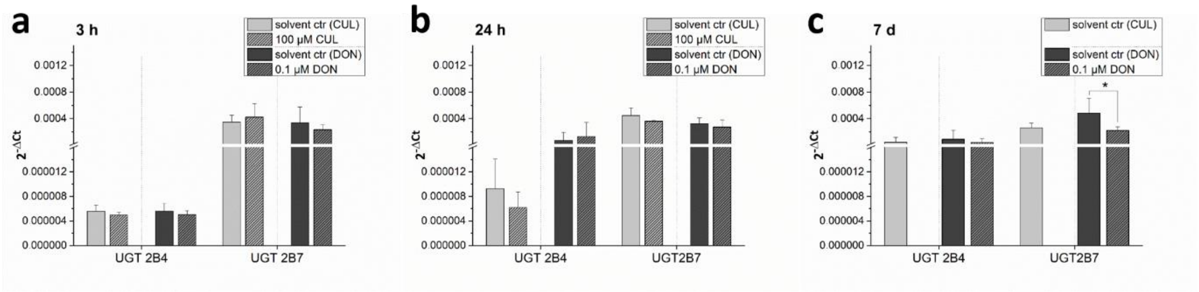

Fig. S5 ***Impact of CUL and DON on gene transcription levels of UGT 2B4 and UGT 2B7 in HT-29 cells after (a) 3 h, (b) 24 h and (c) 7 d of incubation measured by qPCR.*** *Transcription data are normalized to the mean of transcript levels of endogenous control genes. Data are expressed as mean values ± SD of at least three independent experiments performed in technical duplicates. Data were tested by Kruskal-Wallis ANOVA to compare different incubation conditions and * symbols indicate respective significant differences (p<0.05).*

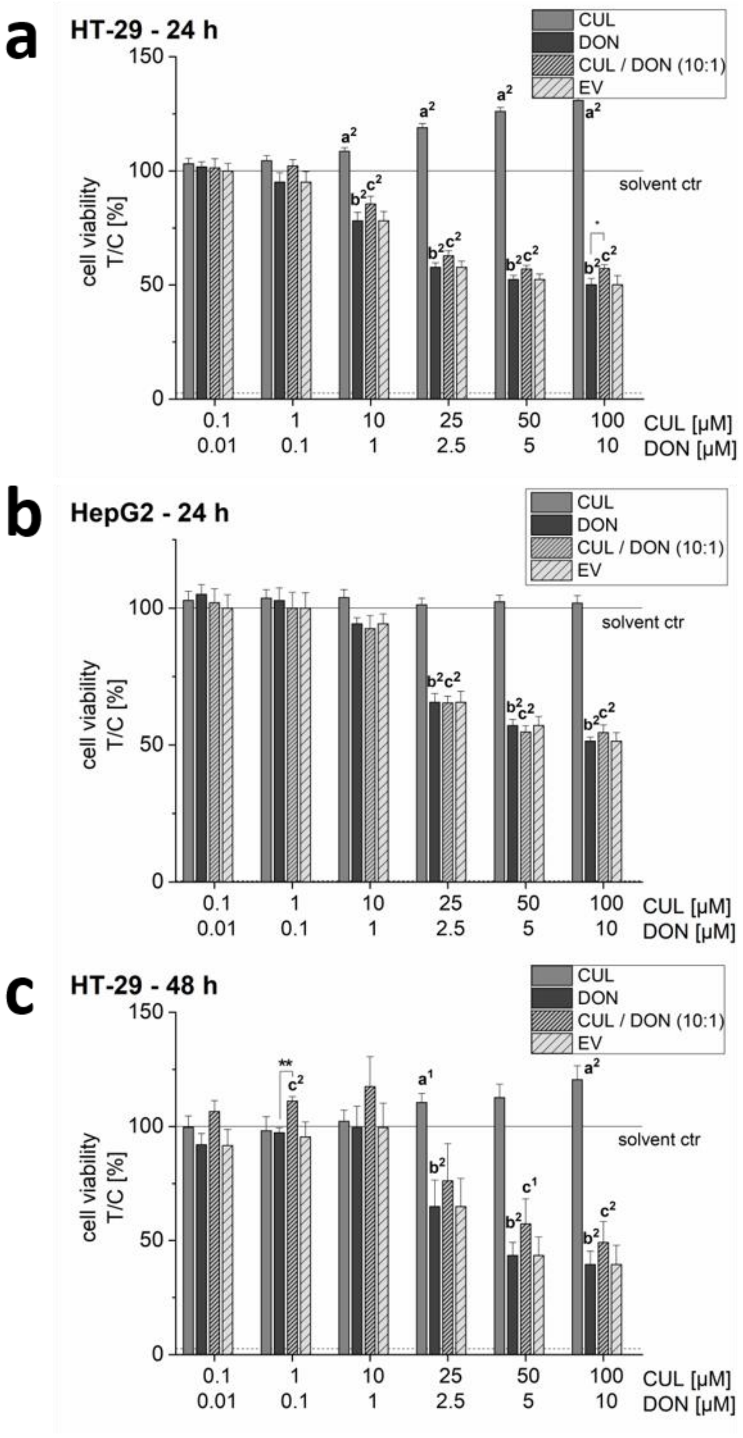

Fig. S6 ***Combinatory effects of CUL with DON on cell viability of HT-29 (a, c) and HepG2 (b) cells in the Sulforhodamine B assay after 24 h (a, b) and 48 h (c) co-incubation.*** *Combinations of CUL and DON were combined 10:1. 1% water (LC-MS grade) + 0.5% DMSO served as solvent controls (solid line). Data are expressed as mean values ± SD of at least five independent experiments performed in triplicates normalized to the respective solvent control. 1% Triton-X 100 was used as positive control (dotted line). Significant differences to the respective lowest tested concentration are indicated in the graphs with (a) for CUL, (b) for DON and (c) for the combination (exponents represent: (1) p<0.05 and (2) p<0.01).*
